# Differences in risk factors for incident and recurrent preterm birth: a population-based linkage of 3.5 million births from the CIDACS birth cohort

**DOI:** 10.1186/s12916-022-02313-4

**Published:** 2022-04-08

**Authors:** Aline S. Rocha, Rita de Cássia Ribeiro-Silva, Rosemeire L. Fiaccone, Enny S. Paixao, Ila R. Falcão, Flavia Jôse O. Alves, Natanael J. Silva, Naiá Ortelan, Laura C. Rodrigues, Maria Yury Ichihara, Marcia F. de Almeida, Mauricio L. Barreto

**Affiliations:** 1 School of Nutrition, Federal University of Bahia, Salvador, Brazil; 2 Center for Data and Knowledge Integration for Health (CIDACS), Oswaldo Cruz Foundation, Salvador, Brazil; 3 Department of Statistics, Federal University of Bahia, Salvador, Brazil; 4 Faculty of Epidemiology and Population Health, London School of Hygiene and Tropical Medicine, London, UK; 5 Institute of Collective Health, Federal University of Bahia, Salvador, Brazil; 6 Barcelona Institute for Global Health, Hospital Clínic, Barcelona, Spain; 7 School of Public Health, University of São Paulo, São Paulo, Brazil

**Keywords:** Preterm birth, Incident preterm birth, Recurrent preterm birth, Risk factor, Poor populations

## Abstract

**Background:**

Preterm birth (PTB) is a syndrome resulting from a complex list of underlying causes and factors, and whether these risk factors differ in the context of prior PTB history is less understood. The aim of this study was to explore whether PTB risk factors in a second pregnancy were different in women with versus without previous PTB.

**Methods:**

We conducted a population-based cohort study using data from the birth cohort of the Center for Data and Knowledge Integration for Health (CIDACS) for the period 2001 to 2015. We used longitudinal transition models with multivariate logistic regression to investigate whether risk factors varied between incident and recurrent PTB.

**Results:**

A total of 3,528,050 live births from 1,764,025 multiparous women were analyzed. We identified different risk factors (*P*_difference_ <0.05) between incident and recurrent PTB. The following were associated with an increased chance for PTB incidence, but not recurrent: household overcrowding (OR 1.09), maternal race/ethnicity [(Black/mixed—OR 1.04) and (indigenous—OR 1.34)], young maternal age (14 to 19 years—OR 1.16), and cesarean delivery (OR 1.09). The following were associated with both incident and recurrent PTB, respectively: single marital status (OR 0.85 vs 0.90), reduced number of prenatal visits [(no visit—OR 2.56 vs OR 2.16) and (1 to 3 visits—OR 2.44 vs OR 2.24)], short interbirth interval [(12 to 23 months—OR 1.04 vs OR 1.22) and (<12 months, OR 1.89, 95 vs OR 2.58)], and advanced maternal age (35–49 years—OR 1.42 vs OR 1.45). For most risk factors, the point estimates were higher for incident PTB than recurrent PTB.

**Conclusions:**

The risk factors for PTB in the second pregnancy differed according to women’s first pregnancy PTB status. The findings give the basis for the development of specific prevention strategies for PTB in a subsequent pregnancy.

**Supplementary Information:**

The online version contains supplementary material available at 10.1186/s12916-022-02313-4.

## Background

Preterm birth (PTB) is one of the main causes of death among children under 5 years of age [1]. PTB is also associated with various complications throughout the lives of survivors as the frequency and severity of adverse outcomes increase as gestational age decreases [2, 3]. In addition, PTB has important economic and social repercussions [4]. The overall rate of PTB has increased from 2000 to 2014 worldwide, and it was estimated at 10.6% in 2014 (14.84 million live births) [5]. In this same year, the proportion of preterm births was 11.2% in Brazil. This figure places the country among the ten countries in the world with the highest rates of PTB [5].

PTB is a syndrome resulting from a complex list of underlying causes and factors including sociodemographic, psychosocial, nutritional, behavioral, and biological factors [6]. Higher rates of PTB are observed in low- and middle-income countries (LMICs) [5], in which socioeconomic disparities have been commonly associated with PTB [7, 8]. Among the risk factors associated with preterm birth is inadequate prenatal care [9]. Adequate prenatal care allows for the diagnosis and treatment of pregnancy complications and a reduction in behavioral risk factors associated with prematurity [10].

Despite this knowledge, whether these risk factors differ in the context of prior preterm birth history is less understood. Also, only one study has distinguished between risk factors for premature birth between women with a previous PTB compared with women without a previous PTB [11]. However, this study was hospital-based from a high-income country with a limited sample size [11]. Using data from the Center for Data and Knowledge Integration for Health (CIDACS) Birth Cohort, we aimed to (1) explore whether the risk factors for PTB in a second pregnancy are different in women whose first pregnancy was delivered at term (≥37 weeks of gestation) or preterm (<37 weeks of gestation) and (2) assess how changes in the number of prenatal visits between pregnancies were associated with PTB in the context of prior PTB.

## Methods

### Study design and population

This population-based cohort study used data from the CIDACS Birth Cohort. This cohort was created by linking data from Brazil’s National Live Birth System (SINASC) and the 100 Million Brazilian Cohort baseline from Jan 1, 2001, to Dec 31, 2015. This study adhered to the RECORD (Reporting of studies Conducted using Observational Routinely-collected Data) statement.

The SINASC gathers information on birth notifications across the country, including information about the mother, pregnancy, newborn, and gestational age at birth, which allows the estimation of the prematurity rate for the country. The live birth declaration forms that provide information to SINASC adopt the last menstrual period (LMP) as the standard method for estimating the gestational age in weeks. Results of physical examinations and other methods are alternatively accepted [12]. The 100 Million Brazilian Cohort is primarily built from Cadastro Único (CadUnico) which covers the poorest half of the Brazilian population (families with monthly income equal to or below three minimum wages ~750 USD) [13]. The CIDACS Birth Cohort is composed of 24,695,617 live births. Children included in the cohort were generally born from younger, unmarried, and less educated mothers and are more likely to be born via vaginal delivery than children in the general Brazilian population [14]. In this study, we identified successive pregnancies using the unique maternal identifier and the newborn’s date of birth.

The linkage process used CIDACS-RL (Record Linkage), a novel record linkage tool developed to link large-scale administrative datasets at CIDACS [15]. The linkage was based on the similarity index using common attributes (mother’s name, maternal age at birth, maternal date of birth, and the municipality of residence of the mother at the time of delivery) between the databases. The linkage process is described in detail elsewhere [16].

**Fig. 1:**
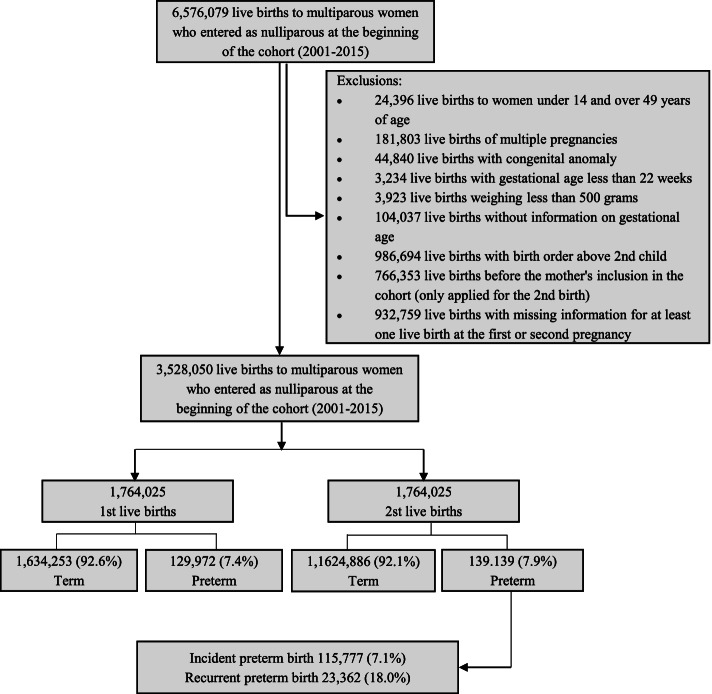
Study population flow diagram

In this study, we included live births of multiparous women, aged 14–49 years, who entered the CIDACS Birth Cohort as nulliparous women. We excluded (a) all multiple births and live births with congenital anomalies as these conditions are known to be strongly associated with premature birth [2, 6], (b) live births weighing < 500 g and gestational age < 22 weeks [17–19], (c) those with a birth date prior to the mother’s entry date into the cohort, and (d) live births with missing information on gestational age and for at least one live birth at the first or second pregnancy (Fig. 1).

### Study variables

The study outcome was the occurrence of PTB in the second pregnancy. Incident PTB was defined as a live birth with a gestational age less than 37 weeks preceded by a term delivery. Recurrent PTB was defined as a live birth with a gestational age less than 37 weeks preceded by a previous PTB.

The following covariates were considered in the analyses: (i) sociodemographic characteristics of the mother: residence area (urban; rural), household overcrowding (no: ≤2 inhabitants per room; yes: >2 inhabitants per room), self-declared race/ethnicity (white, black/mixed-race, or indigenous), level of education (<8 years: 8–11 years; ≥12 years of schooling), marital status (married: married or in a stable relationship; unmarried: single, divorced or widowed); (ii) prenatal assistance: the number of visits (none; 1 to 3 visits; ≥ 4 visits); (iii) maternal, delivery, and newborn-related variables: maternal age (14–19; 20–34; 35–49 years), type of delivery (vaginal or cesarean), and sex of the newborn (female or male), and interbirth interval (<12 months, 12 to 24; ≥24 months). Household overcrowding was calculated by dividing the number of individuals living in the house and the number of rooms. The interbirth interval was estimated (in months) by the difference between the second child's birth date and the previous child’s birth date.

### Statistical analysis

Socioeconomic, maternal, and birth characteristics were summarized using frequency distributions. We used longitudinal transition models with multivariate logistic regression to investigate the risk factors for PTB in a second pregnancy, which is different in women whose first pregnancy was delivered at term (incidence) or preterm (recurrence). Thus, a multiplicative interaction between each incidence and recurrence of PTB was included in the model to assess the risk factors’ chances for incidence versus recurrence in the second pregnancy. Transition models, also called dynamic models or Markov models, are relevant when any response in a sequence of repeated measures is modeled conditionally under one or more sets of past or previous measurements [20]. The specific statistical model for each objective is presented in a footnote for Tables 2 and 3. The transition model used here is a concise form proposed by Diggle et al. [21] to express recurrence and incidence in a single expression as predictors of the previous PTB in the first pregnancy as well as the interactions. The interaction test only identifies risk factors that are different between incidence and recurrence (*α*=0.05). However, the interaction test does not explicitly test whether the risk factor is significant for the incident and recurrent PTB, and thus the odds ratio (OR) and 95% confidence intervals (95% CI) for each risk factor were reported separately according to the first pregnancy PTB status. All models were adjusted for the complete set of risk factors considered to be relevant and plausible in the literature on preterm birth [2, 6, 11, 22–25]. Individuals with missing observations in any of the variables were excluded from the multiple models.

As preventive health behaviors can change between the first and second pregnancies, we assessed the chances of the incident and recurrent PTB according to changes in the number of prenatal visits. We created a four-category variable considering the performance of ≥ 4 prenatal visits in both pregnancies: yes-yes (4 or more appointments in the first and second pregnancy, reference category), yes-no (4 or more appointments in the first and fewer than 4 appointments in the second pregnancy), no-yes (fewer than 4 appointments in the first and 4 or more appointments in the second pregnancy), or no-no (fewer than 4 appointments in the first and second pregnancy). The analyses were adjusted by the same variables mentioned above, except for the number of prenatal visits analyzed as the primary exposure variable.

All analyses were performed using Stata version 15.1 (Stata Corporation, College Station, USA).

### Supplementary analysis

Due to changes in gestational age information in the SINASC registry [26], we conducted complementary analyses with live births born between 2012 and 2015 to verify the consistency of the results of our initial analyses (Additional file 1).

We applied a cross-validation technique using multivariate logistic regression to assess the reliability of our model using. We used a 70% training sample and validated the model with a 30% sample. Receiver operating characteristic (ROC) curves were generated and sensitivity and specificity were calculated for the optimal cut-off based on the ROC curve. The results were compared with those obtained in the general model (Additional file 1).

## Results

During the study period, there were 6,576,079 live births from multiparous women in the CIDACS Birth Cohort. After applying the exclusion criteria, we obtained 3,528,050 (53.7%) live births from 1,764,025 multiparous women. Of this total, 129,772 births (7.4%) were from women who had a PTB in the first pregnancy. At the second pregnancy, 139,139 (7.9%) were PTB, of which 115,777 (7,1%) occurred among 1,634,253 women with a prior term birth (i.e., incident preterm birth), and 23,362 (18%) among the 129,972 women with a previous preterm birth (i.e., recurrent preterm birth) (Fig. 1). Compared to term births, premature births were less frequent among mothers living in rural areas. PTB was more frequent among mothers living in crowded households, single (single, divorced, or widowed), who did fewer than 4 prenatal visits, those with a short interbirth interval, young mothers, and had had a vaginal delivery (Table 1).

**Table 1:** Mother’s sociodemographic characteristics, prenatal care assistance, type of delivery and live birth at second pregnancy by preterm birth status at the first birth, 2001–2015 (*n*=1,764,025)

Second pregnancy variables	Total population (***n*** = 1,764,025)		First pregnancy			
			Term (***n*** = 1,634,253)		Preterm (***n*** = 129,772)	
	*n*	%	*n*	%	*n*	%
**Urban/rural area of residence**						
Urban	1,303,734	77.07	1205,124	76.88	98,604	79.46
Rural	387,896	29.93	362,396	23.12	25,493	20.54
Missing	72,395	4.10				
**Household overcrowding**						
No	1,015,851	62.24	946,688	62.60	69,163	57.67
Yes	616,397	37.76	565,627	37.40	50,770	42.33
Missing	131,777	7.47				
**Maternal race/ethnicity**						
White	541,650	33.66	501,731	33.44	39,919	33.55
Black/mixed-race	1,067,316	65,90	989,219	65.63	78,097	65.63
Indigenous	10,552	0.65	9,564	0.64	988	0.83
Missing	144,507	8.19				
**Maternal education**						
< 8 years of study	638,901	36.78	592,159	36.80	46,742	36.56
8 to 11 years of study	996,380	57.36	921,907	57.29	74,473	58.24
≥ 12 years of study	101,867	5.86	95,220	5.92	6647	5.20
Missing	26,877	1.52				
**Marital status**						
Married, civil union	789,961	45.32	734,464	45.48	55,494	43.28
Single, divorced, widow	953,095	54.68	880,365	54.52	72,729	56.72
Missing	20.969	1.19				
**Number of prenatal visits**						
None	30,255	1.73	27,210	1.68	3045	2.37
1 to 3 visits	154,340	8.81	139,556	8.60	14,784	11.50
≥ 4 visits	1,566,330	89.46	1,455,636	89.72	110,694	86.13
Missing	13,100	0.74				
**Interbirth interval**						
<12 months	20,090	1.14	16,544	1.01	3,546	2.73
12 to 23 months	297,459	16.86	266,622	16.31	30,837	23.76
≥24 months	1,446,476	82.00	1351,087	82.67	95,389	73.50
Missing	0	0.0				
**Maternal age at birth**						
14 to 19 years	325,3112	18.44	290,336	17.77	34,976	26.95
20 to 34 years	1,377,140	78.07	1,286,293	78.71	90,847	70.01
35 to 49 years	61,573	3.49	57,624	3.53	3949	3.04
Missing	0	0.0				
**Type of delivery**						
Vaginal	988,727	56.10	910,214	55.75	78,513	60.53
Cesarean	773,562	43.90	722,428	44.25	51,134	39.44
Missing	1,736	0.10				
**Newborn sex**						
Female	859,905	48.75	796,213	48.72	63,692	49.08
Male	903,963	51.25	837,891	51.28	66,072	50.92
Missing	157	0.01				

We first investigated whether the factors associated with PTB in a second pregnancy differed between women whose first pregnancy was delivered at term or preterm (Table 2). We identified different risk factors (*P*_difference_ <0.05) between incident and recurrent PTB: household overcrowding (OR_incident_ 1.09, 95% CI 1.07–1.10; OR_recurrent_ 1.01, 95% CI 0.98–1.04), maternal race/ethnicity [(Black/mixed—OR_incident_ 1.04, 95% CI 1.03–1.06; OR_recurrent_ 0.95, 95% CI 0.92–0.98) and (indigenous—OR_incident_ 1.34, 95% CI 1.24–1.44; OR_recurrent_ 1.14; 95% CI 0.96–1.35)], single marital status (OR_incident_ 0.85, 95% CI 0.84–0.86; OR_recurrent_ 0.90, 95% CI 0.87–0.93), reduced number of prenatal visits [(1 to 3 visits—OR_incident_ 2.44, 95% CI 2.40–2.49; OR_recurrent_ 2.24; 95% CI 2.14–2.33) and (no visit—OR_incident_ 2.56, 95% CI 2.47–2.66; OR_recurrent_ 2.16; 95% CI 1.98–2.36)], maternal age at birth between 14 and 19 years (OR_incident_ 1.16, 95% CI 1.14–1.18; OR_recurrent_ 1.02, 95% CI 0.98–1.06), and cesarean delivery (OR_incident_ 1.09, 95% CI 1.08–1.11; OR_recurrent_ 0.96, 95% CI 0.92–0.99). For these risk factors, point estimates were higher in magnitude for incident PTB compared to recurrent PTB. Inverse results were observed for short interbirth interval [(12 to 23 months—OR_incident_ 1.04, 95% CI 1.02–1.06; OR_recurrent_ 1.22, 95% CI 1.17–1.26) and (<12 months—OR_incident_ 1.89, 95% CI 1.80–1.98; OR_recurrent_ 2.58, 95% CI 2.38–2.79)] and maternal age between 35 and 49 years (OR_incident_ 1.42, 95% CI 1.38–1.47; OR_recurrent_ 1.45, 95% CI 1.33–1.58). The univariate analyses are presented in Additional file 1: Table S1.

**Table 2:** Frequency and factors associated with incident and recurrent preterm birth in the second pregnancy, 2001–2015 (*n* = 1,764,025)

Second pregnancy variables	Incident preterm birth			Recurrent preterm birth			***P***_**difference**_^**b**^
	*n* (%)	OR^a^	95% CI	*n* (%)	OR^a^	95% CI	
**Urban/rural area of residence**							
Urban	86,747 (7.20)	Ref	Ref	18,003 (18.26)	Ref	Ref	0.96
Rural	24,279 (6.70)	**0.90**	**0.89–0.92**	4395 (17.24)	**0.90**	**0.87–0.94**	
**Household overcrowding**							
No	63,415 (6.70)	Ref	Ref	12,155 (17.57)	Ref	Ref	**<0.00**
Yes	43,080 (7.62)	**1.09**	**1.07–1.10**	9469 (18.65)	1.01	0.98–1.04	
**Maternal race/ethnicity**							
White	33,781 (6.73)	Ref	Ref	7237 (18.13)	Ref	Ref	**<0.00**
Black/Mixed-race	71,741 (7.25)	**1.04**	**1.03–1.06**	14,003 (17.93)	0.95	0.92–0.98	
Indigenous	981 (10.26)	**1.34**	**1.24–1.44**	215 (21.76)	1.14	0.96–1.35	
**Maternal education**							
≥ 12 years of study	5659 (5.94)	Ref	Ref	1,060 (15.95)	Ref	Ref	0.27
8 to 11 years of study	66,183 (7.18)	**1.21**	**1.17–1.25**	13,523 (18.16)	**1.13**	**1.05–1.22**	
<8 years of study	41,944 (7.08) 41,944 (7.08	**1.12**	**1.09–1.16**	8430 (18.04)	1.06	0.98–1.14	
**Marital status**							
Married, civil union	53,922 (7.34)	Ref	Ref	10,132 (18.26)	Ref	Ref	**<0.00**
Single, divorced, widow	60,422 (6.86)	**0.85**	**0.84–0.86**	12,948 (17.80)	**0.90**	**0.87–0.93**	
**Number of prenatal visits**							
≥ 4 visits	90,941 (6.25)	Ref	Ref	17,608 (15.91)	Ref	Ref	**<0.00**
1 to 3 visits	19,606 (14.05)	**2.44**	**2.40–2.49**	4531 (30.65)	**2.24**	**2.14–2.33**	
None	3967 (14.58)	**2.56**	**2.47–2.66**	902 (29.62)	**2.16**	**1.98–2.36**	
**Interbirth interval**							
≥24 months	91,934 (6.80)	Ref	Ref	15,703 (16.46)	Ref	Ref	**<0.00**
12 to 23 months	21,499 (8.06)	**1.04**	**1.02–1.06**	6366 (20.64)	**1.22**	**1.17–1.26**	
<12 months	2344 (14.17)	**1.89**	**1.80–1.98**	1293 (36.46)	**2.58**	**2.38–2.79**	
**Maternal age at birth**							
20 to 34 years	86,081 (6.69)	Ref	Ref	15,415 (16.97)	Ref	Ref	**<0.00**
14 to 19 years	24,548 (8.46)	**1.16**	**1.14–1.18**	7080 (20.24)	1.02	0.98–1.06	
35 to 49 years	5148 (8.93)	**1.42**	**1.38–1.47**	867 (21.95)	**1.45**	**1.33–1.58**	
**Type of delivery**							
Vaginal	64,128 (7.05)	Ref	Ref	14,656 (18.67)	Ref	Ref	**<0.00**
Cesarean	51,494 (7.13)	**1.09**	**1.08–1.11**	8679 (16.97)	0.96	0.92–0.99	
**Newborn sex**							
Female	54,808 (6.88)	Ref	Ref	11,078 (17.39)	Ref	Ref	0.25
Male	60,956 (7.27)	**1.06**	**1.05–1.07**	12,283 (18.59)	**1.08**	**1.05–1.12**	

^a^Analysis adjusted for all model variables

^b^*P*_difference_ represents the interaction between incident and recurrent preterm birth for the respective characteristic. The equation for this model is as follows: Logit [ Pr (PTB2=1/Risk factors)] = β0 + β1 (PTB1) + β2 (risk factor *X*^2^) + β3 (PTB1) × (risk factor *X*^2^), where PTB1 and PTB2 are indicators of a preterm birth in the first and second pregnancy, respectively. Risk factor *X*^2^ is a risk factor in the second pregnancy. The model shows a single risk factor, but additional risk factors were added by including additional terms for each risk factor and its interaction with the first pregnancies’ preterm delivery status, PTB1

We also explored how changes in the number of prenatal visits were associated with the incidence and recurrence of PTB according to the prior delivery history. We observed that live births from mothers with more than four prenatal visits in the first pregnancy who did not have four prenatal visits in the second pregnancy were at increased risk of PTB, mainly if they did not have a previous preterm birth (OR_incident_ 2.57, 95% CI 2.52–2.62; OR_recurrent_ 2.25, 95% CI 2.14–2.37). The chance of incident PTB was 14% higher in the live births from mothers without at least four prenatal visits in the first pregnancy who had this number of visits in the second pregnancy (OR 1.14, 95% CI 1.11–1.18); however, there was no significant increased chance among those recurrent PTB and the reference group. When mothers had an inadequate number of prenatal visits (fewer than four prenatal visits) in both pregnancies, there was a similar increased risk of incident PTB (OR 2.22, 95% CI 2.14–2.30) and recurrent PTB (OR 2.20, 95% CI 2.06–2.38 PTB (Table 3).

**Table 3:** Incident and recurrent preterm birth in the second pregnancy according to change in the number of prenatal visits between the first and the second pregnancies, 2001–2015 (*n* = 1,764,025)

First and second pregnancy variables	Incident preterm birth			Recurrent preterm birth			***P***_**difference**_^b^
	*n* (%)	OR^a^	95% CI	*n* (%)	OR^a^	95% CI	
**≥ 4 prenatal visits**							
Yes-Yes	83,212 (73.35)	Ref	Ref	14,148 (62.19)	Ref	Ref	<0.00
Yes-No	18,762 (16.54)	2.57	2.52–2.62	3324 (14.61)	2.25	2.14–2.37	
No-Yes	6920 (6.10)	1.14	1.11–1.18	3246 (14.27)	1.01	0.96–1.05	
No-No	4553 (4.01)	2.22	2.14–2.30	2033 (8.94)	2.20	2.06–2.38	

^a^Adjusted analysis by mother’s residence area, household overcrowding, mother’s self-declared race/skin color, mother’s level of education, mother's marital status, maternal age, type of delivery in the second birth

^b^*P*_difference_ represents the interaction between incident and recurrent preterm birth for the respective characteristic. The equation for this model is as follows: Logit [ Pr (PTB2=1/Risk factors)] = β0 + β1 (PTB1) + β2 (risk factor *X*^2^) + β3 (PTB1) × (risk factor *X*^2^), where PTB1 and PTB2 are indicators of a preterm birth in the first and second pregnancy, respectively

The analyses restricted to births after 2012 mostly confirmed the findings described above (Additional file 1: Tables S2 and S3). The factors associated with incident and recurrence of preterm birth using the cross-validation technique were similar to those observed in the general model (Additional file 1: Table S4). For incident PTB, we observed accuracy of 50.78% and 50.86% in the training and validation analyses, respectively. For recurrent PTB, we observed accuracy of 52.05% and 51.92% in the training and validation analyses, respectively. The sensitivity and specificity of the models can be seen in Additional file 1: Fig. S1 and S2.

## Discussion

In this study involving 3.5 million live births, we observed different risk factors between incident and recurrent PTB for household overcrowding, maternal race/ethnicity, marital status, number of prenatal visits, maternal age, and delivery mode; association estimates were stronger in magnitude for incident PTB than recurrent PTB. On the other hand, the magnitudes of association for interbirth interval and maternal age were higher for recurrent PTB. The chances of incident and recurrent PTB increased in live births from mothers with fewer than four prenatal visits in the second or in both pregnancies. Four prenatal visits or more in the second pregnancy minimizes the chance of incident PTB.

To our knowledge, risk factors for incident versus recurrent PTB have been reported in a single study [11]. Our findings that a wide range of risk factors was observed for the incident PTB compared to recurrent PTB are consistent with the results reported by Grantz et al. (2014) [11]. Nonetheless, in our study, many risk factors persisted for recurrent PTB after accounting for the excess risk associated with prior PTB history, unlike those reported by the authors above. The mechanisms that explain these results are not fully understood but can be attributed to higher baseline risk among women who have had a previous preterm birth [11]. Another explanation may be related to “index event bias,” which is possible when studying recurrent events. An analysis conditioned to a previous event can induce inverse associations between risk factors (known and unknown). This type of bias can lead to misleading findings and potentially harmful conclusions [27]. We adjusted our analyses for multiple risk factors in an attempt to minimize the index event bias. However, the ability to adjust analyses for all risk factors is a limitation in epidemiological studies in general, and not just in this particular case.

Our results found an increased chance of incident PTB with household overcrowding, Black/mixed and indigenous race/ethnicity, younger ages at birth, and cesarean delivery but not for recurrent PTB. Overcrowding is a marker of poverty and social deprivation [28] and may be associated with PTB since the number of people in a family can influence per capita income, family expenditure, access to food, and other essential services. Crowding can also trigger stress factors on health and well-being, increasing exposure to risk factors for preterm birth [29]. For Black/mixed women, exposure to psychosocial stressors (poverty, homelessness, living in dangerous neighborhoods, domestic violence, experience of discrimination or racism) and risk behaviors associated with stress can favor an increased risk of preterm birth [30]. The association between indigenous race/ethnicity and PTB can be related to worse social and health outcomes of this population when compared to the general population [31]. Concerning the increased risk of PTB among adolescent mothers, some of the explanations proposed are biological immaturity and competition for nutrients between the fetus and the pregnant adolescent [32]. Also, the association between cesarean delivery and preterm birth can be related to the expansion of obstetric interventions aimed at reducing maternal and fetal complications [33].

We observed that a lower number of prenatal visits more than doubled the risk of incident and recurrent PTB. This was notably the strongest factor observed to be associated with PTB incidence. These results corroborate those of several other studies that have reported an increased risk of recurrent PTB among women who make fewer prenatal visits [24, 34]. These findings reinforce the importance of prenatal care for the identification of women at high risk for preterm birth [10]. In our study, we also found lower chances of incident and recurrent PTB among live births from single women (single, widowed, or divorced), with higher protective effect for incident PTB. It is possible that bias in filling out this variable occurred, resulting in the underreporting of women in a stable relationship [35].

We also identified increased chances of incident and recurrent PTB among live births among mothers with short inter-birth intervals and in births from mothers of advanced maternal age, however, unlike the other factors, the estimates were higher for recurrent PTB. Other studies have also identified an increased risk of PTB incident [11] and recurrent [22–25] among women with a short pregnancy interval. This association may be related to maternal nutritional depletion, folate depletion, cervical insufficiency, and infections [36]. Short birth intervals are more common in women from LMICs, where lower socioeconomic status, less education, high fertility rates, and the mother’s age are often associated with short birth intervals [37]. Richer and better-educated women are better off and have access to health services, as well as to information on the use of contraceptive methods and supplies of them, expanding their intervals between deliveries [37]. Preterm birth among women of advanced age may be associated with the increase in clinical complications as age increases, such as arterial hypertension and diabetes mellitus [38].

We also explored changes in the number of prenatal visits between pregnancies and we found that live births from mothers who had a low number of prenatal visits in the first pregnancy and who made four or more prenatal visits in the second pregnancy had less chance of incident PTB, but not recurrent PTB. This finding can be partly explained by the index event bias already discussed in our work and also by Smits et al. [27]. Furthermore, the adoption of ≥4 antenatal visits in the second pregnancy may be affected by the status of the first delivery. The fact is that the first birth status can influence both the follow-up of ≥ 4 prenatal visits during the second pregnancy and the situation of the second birth.

We also observed that the chances of incident and recurrent PTB increased when mothers had fewer than four prenatal visits in the second and both pregnancies. It is known that adequate prenatal care can lead to the adoption of and timely access to preventive measures and effective interventions to reduce biological, social, and behavioral risk factors associated with prematurity [10] in current and subsequent pregnancies. The prevention of premature births occurs through reducing risk behaviors, the identification and treatment of sexually transmitted diseases and other infections, and malnutrition identification and nutritional advice, including supplementation with multiple nutrients [10]. Besides, other health services, such as family planning, favor adequate spacing between pregnancies and reduce the risk of prematurity in subsequent pregnancies [39]. We highlight that, despite the expansion of prenatal care coverage in Brazil in recent decades, there are still regional and social inequalities in access to adequate prenatal care which impact the high level of PTB in our country [40]. Our results reinforce the importance of expanding the access and quality of primary care, especially primary care and access to prenatal care to women in the reproductive phase in order to achieve a reduction in premature births, especially in a current pregnancy.

### Strengths and limitations

This is the first study to assess the factors associated with the incidence and recurrence of PTB in a poor population in a middle-income country with great social and health inequalities. The linkage with the national live birth information system allowed us to track women individually through successive pregnancies. Also, the large number of cases allowed us to simultaneously investigate the factors associated with the incidence and recurrence of PTB, and to carry out our analysis considering the changes in the number of prenatal consultations between pregnancies.

However, this study has some limitations. The use of secondary data, which was not designed primarily for research purposes, may be subject to some limitations related to missing, underestimation, and potential misclassification. The proportion of preterm births recorded in SINASC may be subject to underreporting related to errors in the gestational age [41]. Until 2010, the gestational age at birth was collected over wide intervals of gestational weeks. As of 2011, SINASC started to collect gestational age as a continuous variable; however, the estimation method changed to be mainly based on the LMP [26]. The LMP is the method recommended by WHO, due to its wide accessibility and low cost [42]. Nonetheless, this method can be sometimes not much accurate due to circumstances such as individual variations in the length of the menstrual cycle, especially memory biases [43]. However, these are probably non-differential errors and are unlikely to introduce bias in the measure of association, although the absolute measures of risk may be underestimated. In addition, the proportion of missing data in the cohort could be a limitation for the generalization of our findings. Residual confounding is also possible because important variables for determining PTB, such as maternal comorbidities (e.g., obesity, diabetes, and hypertension), risk behaviors (e.g., smoking, alcohol, or drug use during pregnancy), and access and quality of health services were not available. Also, we were not able to classify the preterm birth subtypes (spontaneous or with medical indication) because of the lack of information in our dataset. The lack of genetic information is another limiting factor; it is known that genetic variants are associated with the duration of pregnancy and the risk of PTB [44]. These limitations may be affecting the differences observed between incident vs. recurrent PTB in this study. In addition, the association between changes in the number of prenatal care visits and PTB according to previous PTB history may present confounding bias. Our findings must be interpreted with caution because the absence of an association between a risk factor and PTB recurrence should not be a reason to dismiss the factor as a potential focus for preventive action. Finally, this study was conducted among the poorest population of a middle-income country with a history of great social and health inequalities which might limit the generalizability of these findings.

## Conclusions

Our study suggests that the risk profiles for PTB in the second pregnancy differ according to women’s first pregnancy PTB status. Household overcrowding, Black/mixed and indigenous race/ethnicity, younger ages at birth, and cesarean delivery were associated with incident PTB but not for recurrent PTB. Incident and recurrent PTB risk factors included single marital status, fewer prenatal visits, short interbirth interval, and advanced maternal age. We found that four or more prenatal visits in the second pregnancy reduce the chance of incident and recurrent PTB. These findings can help to identify women with a high vulnerability to preterm births in a subsequent pregnancy. Furthermore, it can contribute to the development of intervention strategies aimed at reducing this problem and contribute to reducing children’s risk of death.

## Supplementary Information

**Additional file 1: Table S1.** Univariate analyses of factors associated with incident and recurrent preterm birth in the second pregnancy, 2001-2015 (n = 1,764,025). **Table S2.** Frequency and factors associated with incident and recurrent preterm birth in the second pregnancy, 2012-2015 (n = 189,227). ^1^Analysis adjusted for all model variables. ^2^ P_difference_ represents the interaction between incident and recurrent preterm birth for the respective characteristic. The equation for this model is as follows: Logit [ Pr (PTB2=1/Risk factors)] = β0 + β1 (PTB1) + β2 (risk factor X2) + β3 (PTB1) × (risk factor X2), where PTB1 and PTB2 are indicators of a preterm birth in the first and second pregnancy, respectively. **Table S3.** Incident and recurrent preterm birth in the second pregnancy according to change in the number of prenatal visits between the first and the second pregnancies, 2012-2015 (n = 189,482). ^1^Adjusted analysis by mother’s residence area, family density, mother’s self-declared race/skin color, mother’s level of education, mother’s marital status, maternal age, type of delivery in the second birth. ^2^P_difference_ represents the interaction between incident and recurrent preterm delivery for the respective characteristic. The equation for this model is as follows: Logit [ Pr (PTB2=1/Risk factors)] = β0 + β1 (PTB1) + β2 (risk factor X2) + β3 (PTB1) × (risk factor X2), where PTB1 and PTB2 are indicators of a preterm birth in the first and second pregnancy, respectively. **Table S4.** Factors associated with incident and recurrent preterm birth in the second pregnancy, using cross-validation technique, 2001-2015. ^1^Analysis adjusted for all model variables. Figure S1. ROC curve for logistic regression model of incident preterm birth in cross-validation analysis. (A) Empirical ROC curve; (B) Binomial ROC curve. Non-parametric ROC curve not generated due to database size. Figure S2. ROC curve for logistic regression model of recurrent preterm birth in cross-validation analysis. (A) Empirical ROC curve; (B) Binomial ROC curve; (C) Non-parametric ROC curve.

## Data Availability

Data described in the manuscript, code book, and analytic code will be made available upon request.

## Abbreviations

95% CI: 95% confidence interval
CIDACS: Centre for Data and Knowledge Integration for Health
CIDACS-RL: Centre for Data and Knowledge Integration for Health - Record Linkage
ISC-UFBA: Federal University of Bahia’s Collective Health Institute
LMICs: Low- and middle-income countries
LMP: Period of the last menstruation
OR: Odds ratio
PTB: Preterm birth
RECORD: Reporting of studies Conducted using Observational Routinely-collected Data
SINASC: National Live Birth System

## References

[1] United Nations Inter-agency Group for Child Mortality Estimation (UN IGME). Levels & Trends in Child Mortality: Report 2017, Estimates Developed by the UN Inter-agency Group for Child Mortality Estimation. New York: United Nations Children’s Fund; 2017.

[2] Blencowe H, Cousens S, Chou D, Oestergaard M, Say L, Moller AB, et al. Born too soon: the global epidemiology of 15 million preterm births. Reprod Health. 2013;10(Suppl 1):S2. 10.1186/1742-4755-10-S1-S2 PMC3828585 24625129

[3] Mwaniki MK, Atieno M, Lawn JE, Newton CR (2012). Long-term neurodevelopmental outcomes after intrauterine and neonatal insults: a systematic review. Lancet..

[4] March of Dimes, PMNCH, Save the Children, World Health Organization. Born too soon: the global action report on preterm birth born too soon. Eds CP Howson, MV Kinney, JE Lawn. Geneva: World Health Organization; 2012.

[5] Chawanpaiboon S, Vogel JP, Moller A-B, Lumbiganon P, Petzold M, Hogan D (2019). Global, regional, and national estimates of levels of preterm birth in 2014: a systematic review and modelling analysis. Lancet Glob Health.

[6] Goldenberg RL, Culhane JF, Iams JD, Romero R (2008). Epidemiology and causes of preterm birth. Lancet.

[7] Blumenshine P, Egerter S, Barclay CJ, Cubbin C, Braveman PA (2010). Socioeconomic disparities in adverse birth outcomes: a systematic review. Am J Prev Med.

[8] Kramer MS, Seguin L, Lydon J, Goulet L (2000). Socio-economic disparities in pregnancy outcome: why do the poor fare so poorly?. Paediatr Perinat Epidemiol.

[9] Leal MD, Esteves-Pereira AP, Nakamura-Pereira M, Torres JA, Theme-Filha M, Domingues RM (2016). Prevalence and risk factors related to preterm birth in Brazil. Reprod Health.

[10] Requejo J, Merialdi M, Althabe F, Keller M, Katz J, Menon R (2013). Born too soon: care during pregnancy and childbirth to reduce preterm deliveries and improve health outcomes of the preterm baby. Reprod Health.

[11] Grantz KL, Hinkle SN, Mendola P, Sjaarda LA, Leishear K, Albert PS (2015). Differences in risk factors for recurrent versus incident preterm delivery. Am J Epidemiol.

[12] Brasil, Department to Analyze Health Situations, Health. SdVe (2011). Instruction manual to complete the live birth declaration.

[13] Cidacs (2018). Cohort of 100 million Brazilians-2018.

[14] Paixao ES, Cardim LL, Falcao IR, Ortelan N, Silva NJ, Rocha ADS, et al. Cohort profile: the Center for Data and Knowledge Integration for health (CIDACS) birth cohort. Int J Epidemiol. 202050(1):37–8. 10.1093/ije/dyaa255 PMC7938509 33378472

[15] Barbosa GCG, Ali MS, Araujo B, Reis S, Sena S, Ichihara MYT (2020). CIDACS-RL: a novel indexing search and scoring-based record linkage system for huge datasets with high accuracy and scalability. BMC Med Inform Decis Mak.

[16] Almeida D, Gorender D, Ichihara MY, Sena S, Menezes L, Barbosa GCG (2020). Examining the quality of record linkage process using nationwide Brazilian administrative databases to build a large birth cohort. BMC Med Inform Decis Mak.

[17] Upadhyay K, Pourcyrous M, Dhanireddy R, Talati A (2015). Outcomes of neonates with birth weight⩽ 500 g: a 20-year experience. J Perinatol.

[18] Mercer BM (2017). Periviable birth and the shifting limit of viability. Clin Perinatol.

[19] Patel RM, Rysavy MA, Bell EF, Tyson JE (2017). Survival of infants born at periviable gestational ages. Clin Perinatol.

[20] Lordêlo MS, Piedade SMDS, Fernandes GB, Fiaccone RL (2015). Comparação de duas abordagens dos modelos de transição de Markov em experimentos planejados com dados binários correlacionados. Rev Bras Biom.

[21] Diggle P, Diggle PJ, Heagerty P, Liang K-Y, Zeger S. Analysis of longitudinal data. Reino Unido: Oxford University Press; 2013.

[22] Yamashita M, Hayashi S, Endo M, Okuno K, Fukui O, Mimura K (2015). Incidence and risk factors for recurrent spontaneous preterm birth: a retrospective cohort study in Japan. J Obstet Gynaecol Res.

[23] Simonsen S, Lyon J, Stanford J, Porucznik C, Esplin M, Varner M (2013). Risk factors for recurrent preterm birth in multiparous Utah women: a historical cohort study. BJOG Int J Obstet Gynaecol.

[24] Ratzon R, Sheiner E, Shoham-Vardi I (2011). The role of prenatal care in recurrent preterm birth. Eur J Obstet Gynecol Reprod Biol.

[25] Yang J, Baer RJ, Berghella V, Chambers C, Chung P, Coker T (2016). Recurrence of preterm birth and early term birth. Obstet Gynecol.

[26] Brasil, General Coordination of Epidemiological Information and Analyses – CGIAE, Department of Health Surveillance, Health (2013). Consolidação Sistema de informações sobre nascidos vivos: 2011.

[27] Smits LJ, van Kuijk SM, Leffers P, Peeters LL, Prins MH, Sep SJ (2013). Index event bias-a numerical example. J Clin Epidemiol.

[28] Breysse P, Farr N, Galke W, Lanphear B, Morley R, Bergofsky L (2004). The relationship between housing and health: children at risk. Environ Health Perspect.

[29] Ormandy D (2014). Housing and child health. Paediatr Child Health.

[30] Kramer MR, Hogue CJ, Dunlop AL, Menon R (2011). Preconceptional stress and racial disparities in preterm birth: an overview. Acta Obstet Gynecol Scand.

[31] Anderson I, Robson B, Connolly M, Al-Yaman F, Bjertness E, King A (2016). Indigenous and tribal peoples' health (the lancet-Lowitja Institute global collaboration): a population study. Lancet..

[32] Scholl TO, Hediger ML, Schall JI, Khoo CS, Fischer RL (1994). Maternal growth during pregnancy and the competition for nutrients. Am J Clin Nutr.

[33] Morisaki N, Togoobaatar G, Vogel JP, Souza JP, Rowland Hogue CJ, Jayaratne K (2014). Risk factors for spontaneous and provider-initiated preterm delivery in high and low human development index countries: a secondary analysis of the World Health Organization multicountry survey on maternal and newborn health. BJOG Int J Obstetr Gynaecol.

[34] Kistka ZA, Palomar L, Lee KA, Boslaugh SE, Wangler MF, Cole FS (2007). Racial disparity in the frequency of recurrence of preterm birth. Am J Obstet Gynecol.

[35] Gabriel GP, Chiquetto L, Morcillo AM, Ferreira MC, Bazan IGM, Daolio LD (2014). Avaliação das informações das Declarações de Nascidos Vivos do Sistema de Informação sobre Nascidos Vivos (Sinasc) em Campinas, São Paulo, 2009. Revista Paulista de Pediatria.

[36] Conde-Agudelo A, Rosas-Bermudez A, Castano F, Norton MH (2012). Effects of birth spacing on maternal, perinatal, infant, and child health: a systematic review of causal mechanisms. Stud Fam Plan.

[37] Hailu D, Gulte T (2016). Determinants of short Interbirth interval among reproductive age mothers in Arba Minch District, Ethiopia. Int J Reprod Med.

[38] Ludford I, Scheil W, Tucker G, Grivell R (2012). Pregnancy outcomes for nulliparous women of advanced maternal age in South Australia, 1998-2008. Aust N Z J Obstet Gynaecol.

[39] Dean SV, Mason E, Howson CP, Lassi ZS, Imam AM, Bhutta ZA (2013). Born too soon: care before and between pregnancy to prevent preterm births: from evidence to action. Reprod Health.

[40] Domingues RMSM, Viellas EF, Dias MAB, Torres JA, Theme-Filha MM, Gama SGN (2015). Adequacy of prenatal care according to maternal characteristics in Brazil. Rev Panam Salud Publica.

[41] Restrepo-Méndez MC, Lawlor DA, Horta BL, Matijasevich A, Santos IS, Menezes AMB (2015). The association of maternal age with birthweight and gestational age: a cross-cohort comparison. Paediatr Perinat Epidemiol.

[42] WHO (1994). Home-based maternal records : guidelines for development, adaptation and evaluation.

[43] Wegienka G, Baird DD (2005). A comparison of recalled date of last menstrual period with prospectively recorded dates. J Women's Health (Larchmt).

[44] Zhang G, Srivastava A, Bacelis J, Juodakis J, Jacobsson B, Muglia LJ (2018). Genetic studies of gestational duration and preterm birth. Best Pract Res Clin Obstetr Gynaecol.

