# Supplementary material for: Differences in risk factors for incident and recurrent preterm birth: a population-based linkage of 3.5 million births from the CIDACS birth cohort
Source: BMC Med. 2022 Apr 8;20:111. doi: 10.1186/s12916-022-02313-4 (PMC8991880; doi:10.1186/s12916-022-02313-4)
Supplement: Supplementary file 1 — Additional file 1: Table S1. Univariate analyses of factors associated with incident and recurrent preterm birth in the second pregnancy, 2001-2015 (n = 1,764,025). Table S2. Frequency and factors associated with incident and recurrent preterm birth in the second pregnancy, 2012-2015 (n = 189,227). 1Analysis adjusted for all model variables. 2 Pdifference represents the interaction between incident and recurrent preterm birth for the respective characteristic. The equation for this model is as follows: Logit [ Pr (PTB2=1/Risk factors)] = β0 + β1 (PTB1) + β2 (risk factor X2) + β3 (PTB1) × (risk factor X2), where PTB1 and PTB2 are indicators of a preterm birth in the first and second pregnancy, respectively. Table S3. Incident and recurrent preterm birth in the second pregnancy according to change in the number of prenatal visits between the first and the second pregnancies, 2012-2015 (n = 189,482). 1Adjusted analysis by mother’s residence area, family density, mother’s self-declared race/skin color, mother’s level of education, mother’s marital status, maternal age, type of delivery in the second birth. 2Pdifference represents the interaction between incident and recurrent preterm delivery for the respective characteristic. The equation for this model is as follows: Logit [ Pr (PTB2=1/Risk factors)] = β0 + β1 (PTB1) + β2 (risk factor X2) + β3 (PTB1) × (risk factor X2), where PTB1 and PTB2 are indicators of a preterm birth in the first and second pregnancy, respectively. Table S4. Factors associated with incident and recurrent preterm birth in the second pregnancy, using cross-validation technique, 2001-2015. 1Analysis adjusted for all model variables. Figure S1. ROC curve for logistic regression model of incident preterm birth in cross-validation analysis. (A) Empirical ROC curve; (B) Binomial ROC curve. Non-parametric ROC curve not generated due to database size. Figure S2. ROC curve for logistic regression model of recurrent preterm birth in cross-validation analys [file 12916_2022_2313_MOESM1_ESM.docx]

**Differences in risk factors for incident and recurrent preterm birth: a population-based linkage of 3.5 million births from the CIDACS Birth Cohort**

Aline S. Rocha

Online Supplemental Material

**Table S1**: Univariate analyses of factors associated with incident and recurrent preterm birth in the second pregnancy, 2001-2015 (n = 1,764,025)

| **Second pregnancy variables** | **Incident Preterm birth** | | | | | | **Recurrent Preterm Birth** | | |  |  |
| --- | --- | --- | --- | --- | --- | --- | --- | --- | --- | --- | --- |
|  | OR | | 95% CI | | OR | | | 95% CI | | |  |
| **Urban/rural area of residence** | |  | |  | |  | | |  | | |
| Urban | | Ref | | Ref | | Ref | | | Ref | | |
| Rural | | 0.93 | | 0.91 - 0.94 | | 0.93 | | | 0.90 - 0.97 | | |
| **Household overcrowding** | |  | |  | |  | | |  | | |
| No | | Ref | | Ref | | Ref | | | Ref | | |
| Yes | | 1.15 | | 1.13 - 1.16 | | 1.08 | | | 1.04 - 1.11 | | |
| **Maternal race/ethnicity** | |  | |  | |  | | |  | | |
| White | | Ref | | Ref | | Ref | | | Ref | | |
| Black/Mixed-race | | 1.08 | | 1.07 – 1.10 | | 0.99 | | | 0.96 - 1.09 | | |
| Indigenous | | 1.58 | | 1.48 – 1.69 | | 1.27 | | | 1.08- 1.46 | | |
| **Maternal schooling** | |  | |  | |  | | |  | | |
| ≥ 12 years of study | | Ref | | Ref | | Ref | | | Ref | | |
| 8 to 11 years of study | | 1.22 | | 1.19 – 1.26 | | 1.17 | | | 1.09 – 1.25 | | |
| <8 years of study | | 1.21 | | 1.17 – 1.24 | | 1.16 | | | 1.08 – 1.24 | | |
| **Marital status** | |  | |  | |  | | |  | | |
| Married, civil union | | Ref | | Ref | | Ref | | | Ref | | |
| Single, divorced, widow | | 0.94 | | 0.92 – 0.94 | | 0.97 | | | 0.94 – 1.00 | | |
| **Number of prenatal visits** | |  | |  | |  | | |  | | |
| ≥ 4 visits | | Ref | | Ref | | Re, | | | Ref | | |
| 1 to 3 visits | | 2.45 | | 2.41-2.49 | | 2.34 | | | 2.25-2.43 | | |
| None | | 2.56 | | 2.47-2.65 | | 2.23 | | | 2.06-2.41 | | |
| **Inter-birth interval** | |  | |  | |  | | |  | | |
| ≥24 months | | Ref | | Ref | | Ref | | | Ref | | |
| 12 to 23 months | | 1.20 | | 1.20 – 1.22 | | 1.32 | | | 1.28 – 1.36 | | |
| <12 months | | 2.26 | | 2.16 – 2.36 | | 2.91 | | | 2.71 – 3.13 | | |
| **Maternal age birth** | |  | |  | |  | | |  | | |
| 20 to 34 years | | Ref | | Ref | | Ref | | | Ref | | |
| 14 to 19 years | | 1.29 | | 1.27 – 1.31 | | 1.24 | | | 1.20 – 1.28 | | |
| 35 to 49 years | | 1.37 | | 1.33 – 1.41 | | 1.38 | | | 1.27 – 1.49 | | |
| **Type of delivery** | |  | |  | |  | | |  | | |
| Vaginal | | Ref | | Ref | | Ref | | | Ref | | |
| Cesarean | | 1.01 | | 1.00 – 1.02 | | 0.89 | | | 0.87 – 0.92 | | |
| **Newborn sex** | |  | |  | |  | | |  | | |
| Female | | Ref | | Ref | | Ref | | | Ref | | |
| Male | | 1.06 | | 1.05 – 1.07 | | 1.08 | | | 1.05 – 1.12 | | |

**Table S2**: Frequency and factors associated with incident and recurrent preterm birth in the second pregnancy, 2012-2015 (n = 189,227)

| **Second pregnancy variables** | **Incident preterm birth**  **Adjusted analysis*** | | | **Recurrent preterm birth** | | | **P_difference_^2^** ** | | |
| --- | --- | --- | --- | --- | --- | --- | --- | --- | --- |
|  | n (%) | OR^1^ | 95% CI | n (%) | OR* | 95% CI |  | |  |
| **Urban/rural area of residence** |  |  |  |  |  |  |  | |  |
| Urban | 11,443 (9.40) | Ref | Ref | 4,117 (20.56) | Ref | Ref | | 0.03 |  |
| Rural | 3,617 (10.37) | 1.09 | 1.04-1.13 | 1,290 (20.71) | 0.97 | 0.90 – 1.05 | |  |  |
| **Household overcrowding** |  |  |  |  |  |  | |  |  |
| No | 7,144 (9.08) | Ref | Ref | 2,474 (20.36) | Ref | Ref | | 0.11 |  |
| Yes | 7,489 (10.22) | 1.02 | 0.98-1.06 | 2,773 (20.89) | 0.96 | 0.89 - 1.03 | |  |  |
| **Maternal race/ethnicity** |  |  |  |  |  |  | |  |  |
| White | 4,169 (8.77) | Ref | Ref | 1,511 (20.35) | Ref | Ref | | 0.11 |  |
| Black/Mixed-race | 10,045 (9.94) | 1.08 | 1.04 – 1.13 | 3,596 (20.53) | 0.96 | 0.93 – 1.07 | |  |  |
| Indigenous | 208 (15.18) | 1.41 | 1.18 – 1.67 | 88 (25.36) | 1.27 | 0.96-1.67 | |  |  |
| **Maternal schooling** |  |  |  |  |  |  | |  |  |
| ≥ 12 years of study | 576 (7.80) | Ref | Ref | 205 (20.85) | Ref | Ref | | 0.22 |  |
| 8 to 11 years of study | 5,107 (11.03) | 1.01 | 0.98 – 1.21 | 1,852 (20.93) | 0.85 | 0.71– 1.03 | |  |  |
| <8 years of study | 9,107 (9.07) | 1.09 | 0.92-1.12 | 3,403 (20.15) | 0.82 | 0.68– 0.99 | |  |  |
| **Marital status** |  |  |  |  |  |  | |  |  |
| Married, civil union | 7,309 (9.24) | Ref | Ref | 2,536 (19.83) | Ref | Ref | | 0.37 |  |
| Single, divorced, widow | 8,092 (9.92) | 0.99 | 0.96 – 1.03 | 2,952 (21.05) | 1.03 | 0.97 – 1.10 | |  |  |
| **Number of prenatal visits** |  |  |  |  |  |  | |  |  |
| ≥ 4 visits | 10,907 (8,06) | Ref | Ref | 3,736 (17.41) | Ref | Ref | |  |  |
| 1 to 3 visits | 3,791 (17.23) | 2.10 | 2.01- 2.20 | 1,468 (32.40) | 2.14 | 1.97 – 2.31 | | 0.00 |  |
| None | 669 (17.53) | 2.16 | 1.96 - 2.37 | 262 (31.64) | 1.63 | 1.98 – 2.28 | |  |  |
| **Inter-birth interval** |  |  |  |  |  |  | |  |  |
| ≥24 months | 6,343 (8.01) | Ref | Ref | 1,998 (16.79) | Ref | Ref | | 0.03 |  |
| 12 to 23 months | 8,269 (10.58) | 1.22 | 1.17 – 1.27 | 2,955 (21.64) | 1.29 | 1.20 – 1.39 | |  |  |
| <12 months | 955 (18.84) | 2.30 | 2.12 – 2.50 | 596 (38.43) | 2.77 | 2.44 – 3.13 | |  |  |
| **Maternal age birth** |  |  |  |  |  |  | |  |  |
| 20 to 34 years | 8,621 (8.46) | Ref | Ref | 2,771 (19.04) | Ref | Ref | | <0.00 |  |
| 14 to 19 years | 6,758 (11.58) | 1.21 | 1.16 – 1.26 | 2,709 (22.13) | 1.03 | 0.96 – 1.11 | |  |  |
| 35 to 49 years | 188 (8.86) | 1.25 | 1.06 – 1.48 | 69 (22.12) | 1.33 | 0.99 – 1.80 | |  |  |
| **Type of delivery** |  |  |  |  |  |  | |  |  |
| Vaginal | 9,439 (10.46) | Ref | Ref | 3.773 (22.12) | Ref | Ref | | 0.01 |  |
| Cesarean | 6,096 (8.46) | 0.90 | 0.87 – 0.94 | 1,768 (17.66) | 0.81 | 0.75 – 0.87 | |  |  |
| **Newborn sex** |  |  |  |  |  |  | |  |  |
| Female | 7,414 (9.40) | Ref | Ref | 2,585 (19.60) | Ref | Ref | | 0.03 |  |
| Male | 8,152 (9.76) | 1.04 | 1.01 – 1.09 | 2,964 (21.29) | 1.14 | 1.07 – 1.22 | |  |  |

^1^Analysis adjusted for all model variables.

^2^ P_difference_ represents the interaction between incident and recurrent preterm birth for the respective characteristic. The equation for this model is as follows: Logit [ Pr (PTB2=1/Risk factors)] $=$ β0 + β1 (PTB1) + β2 (risk factor X2) + β3 (PTB1) × (risk factor X2), where PTB1 and PTB2 are indicators of a preterm birth in the first and second pregnancy, respectively.

| **Table S3**: Incident and recurrent preterm birth in the second pregnancy according to change in the number of prenatal visits between the first and the second pregnancies, 2012-2015 (n = 189,482) |
| --- |

| **First and second pregnancy variables** | **Incident Preterm birth** | | | **Recurrent Preterm birth** | | | | **P_difference_^2^** |
| --- | --- | --- | --- | --- | --- | --- | --- | --- |
|  | n (%) | OR^1^ | 95% CI | n (%) | OR^1^ | | 95% CI |  |
| **≥ 4 prenatal visits** |  |  |  |  |  | |  |  |
| Yes-Yes | 9,967 (65.27) | Ref | Ref | 2,908 (53.73) | Ref | Ref | | 0.30 |
| Yes-No | 3,496 (22.89) | 2.17 | 2.07-2.27 | 1,052 (19.44) | 2.20 | 2.00-2.40 | |  |
| No-Yes | 885 (5.80) | 1.20 | 1.10-1.29 | 792 (14.63) | 1.27 | 1.16-1.40 | |  |
| No-No | 922 (6.04) | 2.04 | 1.88-2.21 | 660 (12.20) | 2.31 | 2.06-2.58 | |  |

^1^Adjusted analysis by mother's residence area, family density, mother's self-declared race/skin color, mother's level of education, mother's marital status, maternal age, type of delivery in the second birth.

^2^P_difference_ represents the interaction between incident and recurrent preterm delivery for the respective characteristic. The equation for this model is as follows: Logit [ Pr (PTB2=1/Risk factors)] $=$ β0 + β1 (PTB1) + β2 (risk factor X2) + β3 (PTB1) × (risk factor X2), where PTB1 and PTB2 are indicators of a preterm birth in the first and second pregnancy, respectively.

**Table S4**: Factors associated with incident and recurrent preterm birth in the second pregnancy, using cross-validation technique, 2001-2015

| **Second pregnancy variables** | **Incident preterm birth** | | **Recurrent preterm birth** | |
| --- | --- | --- | --- | --- |
|  | OR^1^ | 95% CI | OR^1^ | 95% CI |
| **Urban/rural area of residence** |  |  |  |  |
| Urban | Ref | Ref | Ref | Ref |
| Rural | 0.91 | 0.90 – 0.93 | 0.91 | 0.86 – 0.95 |
| **Household overcrowding** |  |  |  |  |
| No | Ref | Ref | Ref | Ref |
| Yes | 1.09 | 1.08 – 1.11 | 1.01 | 0.97 – 1.05 |
| **Maternal race/ethnicity** |  |  |  |  |
| White | Ref | Ref | Ref | Ref |
| Black/Mixed-race | 1.05 | 1.03 – 1.07 | 0.95 | 0.91 – 0.99 |
| Indigenous | 1.31 | 1.19 – 1.43 | 1.08 | 0.87 – 1.32 |
| **Maternal schooling** |  |  |  |  |
| ≥ 12 years of study | Ref | Ref | Ref | Ref |
| 8 to 11 years of study | 1.11 | 1.07 – 1.15 | 1.05 | 0.97 – 1.16 |
| <8 years of study | 1.20 | 1.16 – 1.25 | 1.13 | 1.04 – 1.24 |
| **Marital status** |  |  |  |  |
| Married, civil union | Ref | Ref | Ref | Ref |
| Single, divorced, widow | 0.84 | 0.83 – 0.86 | 0.90 | 0.86 – 0.93 |
| **Number of prenatal visits** |  |  |  |  |
| ≥ 4 visits | Ref | Ref | Ref | Ref |
| 1 to 3 visits | 2.43 | 2.38 – 2.49 | 2.30 | 1.92 – 2.37 |
| None | 2.57 | 2.45 – 2.69 | 2.13 | 1.92 – 2.37 |
| **Inter-birth interval** |  |  |  |  |
| ≥24 months | Ref | Ref | Ref | Ref |
| 12 to 23 months | 1.03 | 1.02 – 1.06 | 1.22 | 1.16 – 1.28 |
| <12 months | 1.91 | 1.8-0 – 2.03 | 2.64 | 2.39 – 2.90 |
| **Maternal age birth** |  |  |  |  |
| 20 to 34 years | Ref | Ref | Ref | Ref |
| 14 to 19 years | 1.15 | 1.13 – 1.18 | 1.00 | 0.96 – 1.05 |
| 35 to 49 years | 1.41 | 1.35 – 1.46 | 1.45 | 1.31 – 1.61 |
| **Type of delivery** |  |  |  |  |
| Vaginal | Ref | Ref | Ref | Ref |
| Cesarean | 1.09 | 1.08 – 1.11 | 0.97 | 0.93 – 1.01 |
| **Newborn sex** |  |  |  |  |
| Female | Ref | Ref | Ref | Ref |
| Male | 1.06 | 1.05 – 1.08 | 1.08 | 1.04 – 1.13 |

^1^Analysis adjusted for all model variables.

B

A


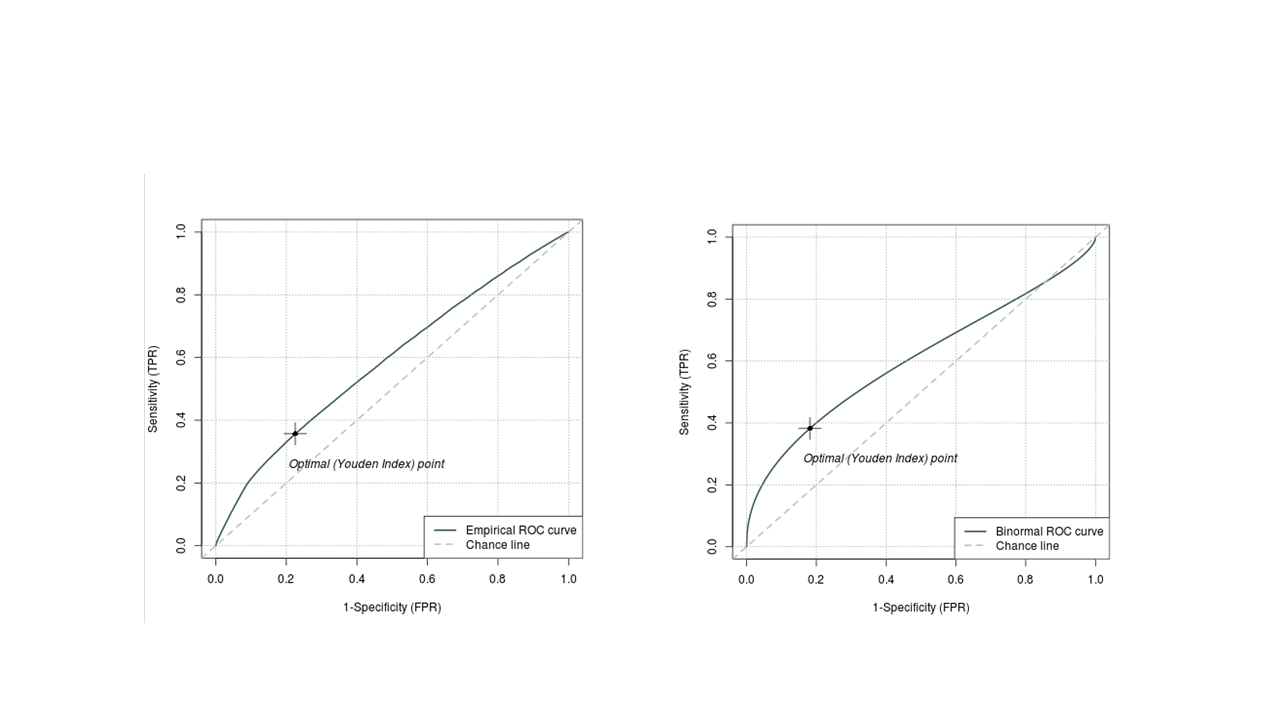


**Figure S1**. ROC curve for logistic regression model of incident preterm birth in cross-validation analysis. (A) Empirical ROC curve; (B) Binomial ROC curve.

Non-parametric ROC curve not generated due to database size.

C

B

A


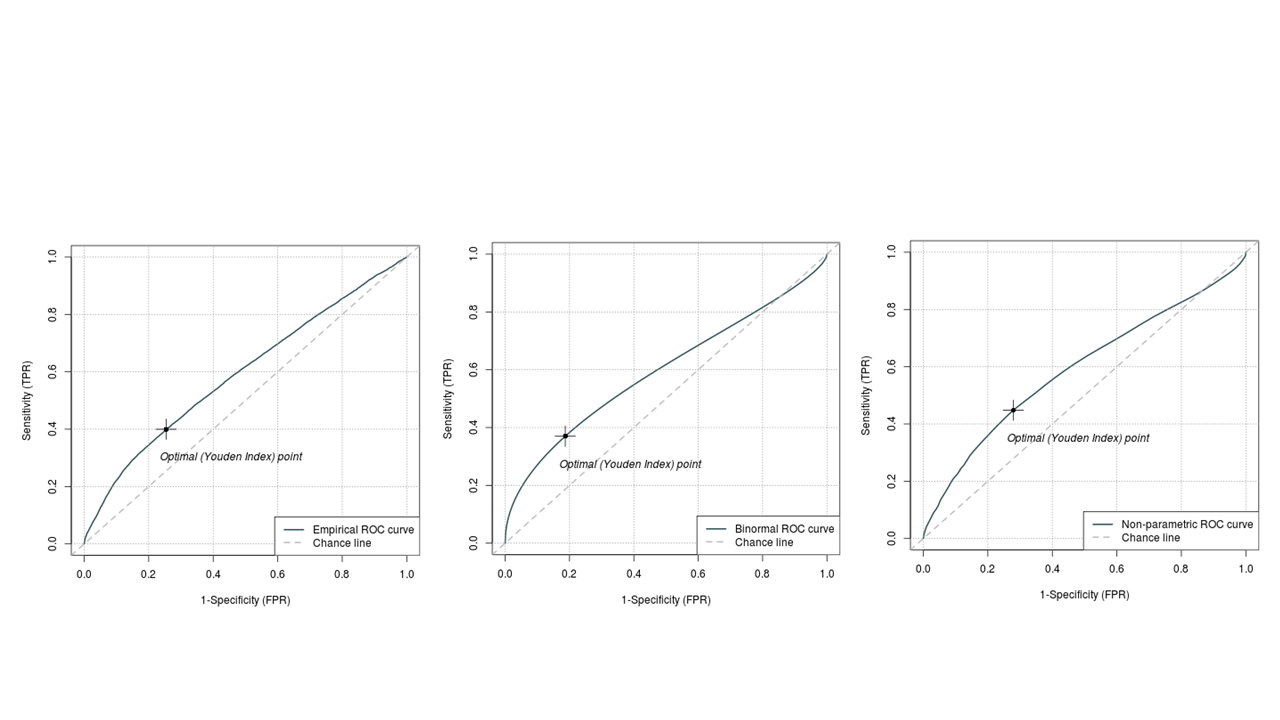


**Figure S2**. ROC curve for logistic regression model of recurrent preterm birth in cross-validation analysis. (A) Empirical ROC curve; (B) Binomial ROC curve; (C) Non-parametric ROC curve.
